# Clinic of Prof. N. S. Davis—Summer Complaints of Children—Cases, &c.

**Published:** 1872-06-15

**Authors:** 


### Clinical Reports.

### CLINIC OF PROF. N. S. DAVIS—SUMMER COMPLAINTS OF CHILDREN—CASES, &c.

*Gentlemen:* In the cities and more densely populated towns located in the middle and northern belts of the United States, the first week of continuous hot summer weather always develops more or less cases of diarrhœa and cholera-morbus in young children, and to a less degree also in adults. In this city (Chicago), the time of commencing the high summer heat is very variable, but generally manifests itself between the twenty-third of June and the middle of July. In each of the five years that I have witnessed the prevalence of epidemic cholera here, the first week of high temperature commenced during the last week of June. In 1854, we had a week of hot, sultry weather, commencing as early as the twenty-first of June. The present season, we had a succession of three very hot, oppressive days, between the twentieth and twenty-fourth of June, ending in a copious rain, with two or three cooler days. From that to the present time (July 2d) we have had continuous hot weather, with oppressive south and south-west winds, inducing a great feeling of lassitude in persons in good health.

During the last of the first three days, I was called to several cases of diarrhœa and vomiting in children, and to two cases of cholera-morbus, accompanied by muscular cramps, in adults. During the last four days the attacks of diarrhœa, cholera-infantum and cholera-morbus, have increased so rapidly, that nearly one-half of all the patients coming under my observation have been of that class; and the past week's mortality will show a large increase over the previous weeks, from the prevalence of these affections. This large increase of mortality, chiefly from the bowel-affections of children, recurs regularly every year, beginning the first week of hot summer weather and continuing through July, August, and a part of September; and these affections are as strictly endemic in the impure summer atmosphere of the cities in the temperate zone of the earth's surface, as the intermittent fever is on the Roman Campagna or on the alluvial deposits in the Mississippi Valley. The principal determining causes appear to be a high temperature acting in conjunction with an atmosphere either deficient in free electricity and ozone, or rendered impure by the products of animal and vegetable decomposition.

These influences induce a great degree of relaxation or diminution of vital affinity, with a morbidly sensitive condition of the mucous and cutaneous surfaces of the body, which, in the more feeble organization of very young children, is sufficient to cause an exudation from the mucous membranes and its ejection by vomiting or purging, or both. If the relaxation is excessive and the exudation or effusion from the mucous surfaces rapid, causing active vomiting and purging, it takes the name of cholera-morbus or cholera-infantum; when it is slight, causing only languor, paleness, and several thin discharges from the bowels, it takes the name of diarrhœa or "summer complaint."

Case 1st.—Here is a case illustrating the last mentioned class. This baby is eight months old. You see it lying languidly in its mother's lap; the face is a little pale; the eye slightly sunken; the expression sad; its surface and extremities cool; respiration quiet, and pulse soft and weak. The mother says it has had from four to six thin, yellow discharges every twenty-four hours for the last three days. The passages are thin, copious, and preceded by a little restlessness or peevishness, and followed by languor. There is neither fever, pain, or mucus in the discharges, or anything indicating local inflammatory action. The pathological conditions are, simply, general relaxation, with undue excitability of the mucous membranes of the bowels. The ideas entertained by some, that these cases depend on some derangement of the liver; or that the discharges are the result of an effort of nature to get rid of some morbid matter, or the result of "teething," are founded on neither legitimate reasoning nor the facts involved.

The indications for the treatment of the case before you are, to allay the morbid excitability of the mucous membranes and give increased tone to the nervous and muscular structures of the body. The child's nursing should be regulated so as to prevent overloading the stomach at any one time, but the mother's milk is the best nourishment that it can take, and the less of any other fluid it takes the better.

For medicine we will give it the following prescription:

| | |
|---|---|
| ℞—Phloridzine | 24 grains. |
| Aromat. spts. ammon. | ʒ i. |
| Camph. tinct. opii | ℥ i. |
| Water | ℥ iss. |
| Simple syrup | ℥ ss. |

Mix. Shake the vial and give half a teaspoonful each morning, noon, teatime, and at bedtime.

The phloridzine, derived from the bark of the root of the appletree, is a mild and pleasant tonic, while the camphorated tincture of opium supplies the necessary anodyne influence. In mild cases, like the one before us, this combination has been frequently used with prompt benefit.

Case 2d. — This is a child presenting another aspect of the same general malady. Its mother says it is thirteen months old, and was suddenly attacked last evening, with vomiting and purging, every few minutes until the present time. If it nurses, the milk is ejected almost as soon as it lets go the nipple; if it drinks a spoonful of water it provokes the same heaving; and thus everything it swallows is speedily thrown up. Yet it is craving for water. The passages from the bowels occur every half-hour, perhaps, and are preceded by a little writhing and fretting, and consist of a turbid or yellowish fluid, so thin as to run readily through two or three napkins, and leave only a stain of fecal matter. The child is drowsy; the eyes sunken; the face pale and dejected; the skin, especially of the extremities, cool; pulse thready and weak; and respirations slow, with frequent sighing. Here you have a more active example of the cholera-infantum, about eighteen hours after the commencement of the attack.

It is a case presenting actual danger of fatal collapse during the active stage. If it survives this stage, and the vomiting becomes only occasional, with a continuance of intestinal discharges less frequently, the latter will become green, mixed with little masses of mucus, sometimes streaked with blood; the abdomen will become hot, the pulse quick, the child more restless and peevish, with excessive thirst, and after emaciating to a skeleton, may die from inanition at the end of three, four or six weeks. On the other hand, prompt treatment designed to allay the extreme irritability of the whole extent of the mucous surface of the alimentary canal, and restore the proper tone of the capillary vessels, will in many cases arrest the disease and lead to a rapid recovery of the patient. We will give the little sufferer the following prescriptions:

| | |
|---|---|
| ℞—Carbolic acid crystals | 3 grains. |
| Glycerine (pure) | ℥ ss. |
| Camph. tinct. opii | ℥ i. |
| Water | ℥ iss. |

Mix. Give 20 drops every half hour until the vomiting ceases; then extend the time to every two hours.

| | |
|---|---|
| ℞—Hydrg. chlorid. mite | 4 grains. |
| Pulv. opii | 1 " |
| White sugar | 30 " |

Mix. Divide into 8 powders, and give one every 8 hours.

If the vomiting ceases, and the passages from the bowels are reduced in frequency to only two or three in the twenty-four hours, the powders are to be omitted, and ten drops of the nitrous ether added to each dose of the carbolic acid solution, to aid in securing proper action of the kidneys. In many cases, this treatment will result in a rapid and entire recovery.

In others, the vomiting ceases, or occurs only occasionally; but the intestinal discharges continue at intervals of two, three, or four hours, preceded and accompanied by symptoms of pain; the child becomes fretful, craving for drink, and rapidly emaciates. The intestinal discharges are very variable in color and quality.

In a majority of such cases the following emulsion acts very favorably:

| | | |
|---|---|---|
| ℞—Ol. terebinth | | ʒ ii. |
| Ol. wintergreen | | 20 gtts. |
| Tinct. opii | | ʒ ii. |
| Pulv. G. Arabic | a a | ʒ iv. |
| White sugar | | |
| Rub together and add | | |
| Water | | ℥ iii. |

Mix. Shake the vial and give to a child of this age, from fifteen to twenty drops every three, four, or six hours, according to the frequency of the discharges. In all cases where the child can have a good breast of milk, that alone should be its nourishment. But if artificial food must be provided, we have found nothing to answer better than a thin, well-prepared wheat-flour and milk porridge, given in small quantities. It is also of great importance to give these little children access to fresh and pure air. Their confinement in small, over-heated and badly ventilated rooms, is one of the most prolific causes of their sickness and mortality.

Hydrochlorate of Narceine for Hypodermic Injections.—Dr. Petrini, in the *Bulletin de Therap.*, advocates this salt as superior to sulphate of atropine or muriate of morphia. In very small doses, five milligr. to one centigr., its calmative power is manifest. It is superior in its power to control sickness to salts of morphia. When all preparations of opium or morphia fail as hypnotics, a small dose of the salt of narceine will succeed. In the smallest doses its effects are also to slightly raise the temperature, as well as the frequency of both the pulse and respiration, but at the same time to reduce arterial tension.—*Medical World.*

Croup.—MM. Mouttet and Rosiere, of Montpellier, report that they have cured croup, when tracheotomy was refused by the parents as a useless cruelty, by repeated insufflations of powdered nitrate of silver. The application was at once followed by the detachment and expulsion of false membrane, and was repeated a quarter of an hour after with a similar result. Three or four hours later another insufflation was followed by so much relief that hope was entertained, and the child from this time made a steady recover.—*Id.*

Intussusception—Novel and Successful Treatment.—Alfred W. Taliaferro, of San Rafael, reports a case of invagination of the intestines that was relieved, after all other means had failed, by an injection of a solution of carbonate of soda followed by a solution of tartaric acid. Immediately on the withdrawal of the pipe of the syringe, after the injection of the second solution, a large sponge was pressed against the anus.—*Western Lancet.*

